# Activity-Dependent Neuroprotective Protein (ADNP): An Overview of Its Role in the Eye

**DOI:** 10.3390/ijms232113654

**Published:** 2022-11-07

**Authors:** Grazia Maugeri, Agata Grazia D’Amico, Benedetta Magrì, Giuseppe Musumeci, Velia D’Agata

**Affiliations:** 1 Section of Anatomy, Histology and Movement Sciences, Department of Biomedical and Biotechnological Sciences, University of Catania, 95123 Catania, Italy; 2 Department of Drug Sciences, University of Catania, 95123 Catania, Italy

**Keywords:** ADNP, NAP, eye, anatomy

## Abstract

Vision is one of the dominant senses in humans and eye health is essential to ensure a good quality of life. Therefore, there is an urgent necessity to identify effective therapeutic candidates to reverse the progression of different ocular pathologies. Activity-dependent neuroprotective protein (ADNP) is a protein involved in the physio-pathological processes of the eye. Noteworthy, is the small peptide derived from ADNP, known as NAP, which shows protective, antioxidant, and anti-apoptotic properties. Herein, we review the current state of knowledge concerning the role of ADNP in ocular pathologies, while providing an overview of eye anatomy.

## 1. Introduction

Activity-dependent protein (ADNP) is a neuroprotective protein of 123.56 kDa molecular weight, widely expressed throughout the body, including the eye. Morphological and proteomic studies showed that ADNP is distributed in the retina and cornea of different species, including humans [1,2]. ADNP was originally discovered as an astroglial secreted protein, able to modulate the neurotrophic/neuroprotective activity of vasoactive intestinal peptide (VIP), as well as of pituitary adenylate cyclase-activating peptide (PACAP) [3,4]. PACAP and VIP perform their effects through the activation of G protein-coupled receptors, pituitary adenylate cyclase-activating polypeptide receptor type 1 (PAC1R), vasoactive intestinal polypeptide receptor 1 (VPAC1R), and vasoactive intestinal polypeptide receptor 2 (VPAC2R). In particular, the PAC1 receptor shows eight different splice variants (Null, Hip, Hop1, Hop2, Hiphop1, Hiphop2, short, and very short isoforms), whose activation by the binding to PACAP/VIP activates phospholipase C (PLC) and adenylate cyclase (AC), or calcium-regulated mechanisms [5]. It is worth noting that a subpicomolar concentration of PACAP stimulated ADNP expression mainly through the MAPK signaling pathway and cAMP-dependent protein kinase activation [6]. Both VIP and PACAP showed important protective effects against different ocular diseases [7,8,9,10,11,12,13,14,15,16,17]. The therapeutic use of PACAP or VIP presents some limitations, due to their short half-life caused by rapid enzymatic degradation [18,19].

NAP (davunetide, NAPVSIPQ/Asn-Ala-Pro-Val-Ser-Ile-Pro-Gln), the short peptide derived from the ADNP sequence, demonstrated comparable protective effects in the eye with a longer half-life, as compared to PACAP and VIP. Moreover, clinical trials conducted with NAP for progressive supranuclear palsy, mild cognitive impairment, and schizophrenia showed safety and tolerance in hundreds of adult patients [20].

The present review provides an overview on the eye’s anatomy and summarizes data present in literature regarding the role of ADNP in the eye, particularly in the retina and cornea, by hypothesizing the possibility of its clinical application.

## 2. Overview of Eye Anatomy

**Figure 1:**
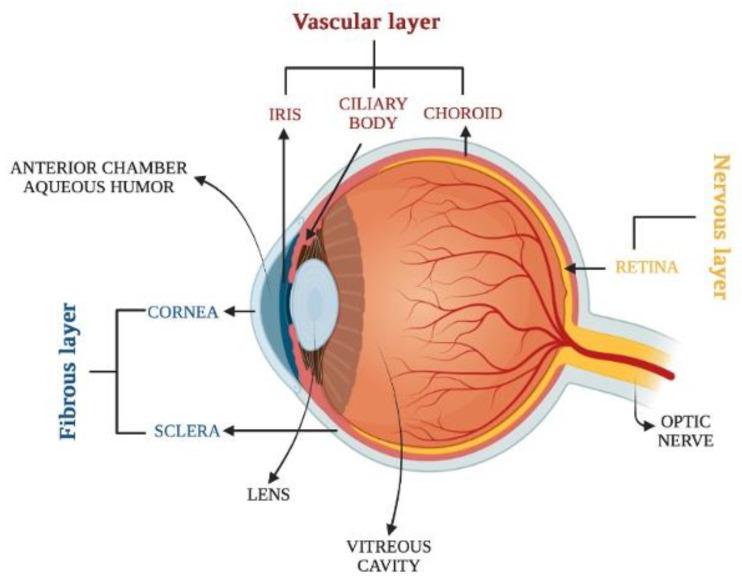
The basic anatomy of the human eye.

The human eye is able to percept a multitude of shapes and colors with a resolution of 576 gigapixels. This extraordinary ability is facilitated by the complex structure of the eyeball, which comprises three distinct layers: the fibrous layer, the vascular layer and the nervous layer (Figure 1).

The fibrous tunic is the outermost layer and is composed of the sclera and the cornea. The sclera supports the wall and the shape of the eyeball protects it from injury. It is formed by dense connective tissue, whose type 1 collagen fibers are oriented in different directions, giving the sclera a white appearance [21]. The cornea covers the anterior portion of the eyeball. It is mechanically strong and transparent and provides about 70% of the eye’s refractive power [22]. The cornea is formed by five main layers: the epithelium, the Bowman’s membrane (BM), the stroma, the Descemet’s membrane (DM) and the endothelium. The epithelium is characterized by ~six layers of nonkeratinized squamous epithelial cells, morphologically distinguished into the basal columnar, wing, and superficial squamous cells. The human BM has a thickness ranging from 8 to 12 μm and is formed by collagen fibrils involved in the maintenance of the corneal shape [23]. The stroma, representing 80% of corneal thickness, is a fibrous, tough and transparent layer, composed of 200 flattened lamellæ overlapped with collagen fibrils. The keratocytes represent the main stromal cells regulating stromal homeostasis through the synthesis of collagen, glycosaminoglycans, and matrix metalloproteinases [24]. The DM is a dense and acellular matrix located between the stroma and the endothelium formed by Type IV collagen and laminin. The innermost layer of the cornea is the endothelium, formed by a single layer of flat cells with a hexagonal shape, acting as a barrier and functional pump. The corneal endothelial cells have limited proliferative capability in vivo since they are arrested in the G1 phase of the cell cycle due to contact inhibition [25]. The vascular layer, known as the uvea, is distinguished into three anatomical parts. Proceeding from the posterior to the anterior region of the eyeball, it includes the choroid, ciliary body, and iris. The choroid, characterized by a dense network of blood vessels, ensures a constant supply of nourishment to structures of the eye localized in loose connective tissue. The ciliary body is involved in aqueous humor formation. Moreover, the base of this structure is home to the ciliary muscle, whose contraction changes the curvature of the lens leading to a process known as accommodation [26]. The iris is a circular, pigmented diaphragm, which divides the space between the cornea and the lens into the anterior and posterior chambers, both containing the aqueous humor. Through the contraction of the pupil sphincter or dilator muscle, the iris regulates the amount of light that penetrates the eye [27]. The nervous layer is the retina, representing a functional part of the central nervous system, able to convert the light signal into action potentials arriving in the brain through the optic nerve (ON). The retina comprises five types of neurons: the photoreceptors (cones and rods), the horizontal cells, the bipolar cells, the amacrine cells and the ganglion cells. The soma of these neurons is localized in the photoreceptors layer (RCL), outer nuclear layer (ONL), inner nuclear layer (INL) and ganglion cell layer (GCL), respectively. Instead, the processes and synaptic contacts are placed in the outer plexiform layer (OPL) and the inner plexiform layer (IPL). The retina also comprises three types of glial cells, i.e., astrocytes, microglial cells and Müller glial cells. The latter can regenerate retinal neurons exposed to different insults by providing nutrients and trophic factors [28]. The outer part of the retina is formed by the pigmented epithelial (RPE) cells, involved in tissue homeostasis, since they regulate the transport of molecules from the choroid to the sub-retinal area and the elimination of waste products [29]. Moreover, the RPE cells contribute to maintaining the integrity of the blood–retinal barrier and convert all-trans-retinol to 11-cis-retinal needed for light perception [30].

Vision begins in the retina when the light crosses its thickness and activates the rods and cones. The latter converts the light into an electrical signal, passing the information to the horizontal, bipolar, amacrine cells and, in the end, to the retinal ganglion cells (RGCs), whose axons form the optic nerve which propagates the visual stimulus from the eye to the brain [31]. The health and proper functioning of all ocular structures are necessary to ensure a well-defined vision. Diabetic retinopathy (DR), cataract, glaucoma, uncorrected refractive error and age-related macular degeneration represent the main causes of serious vision impairment. Therefore, the research is aimed at identifying new therapeutic targets to prevent and counteract blindness resulting from these diseases [32].

## 3. Activity-Dependent Neuroprotective Protein (ADNP): Expression and Functions

**Figure 2:**
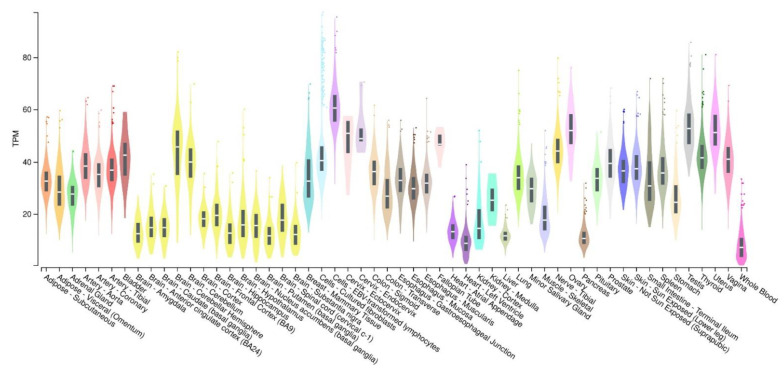
Boxplot of transcripts per million (TPM) showing the bulk tissue gene expression for ADNP. GTEx Portal on 29 September 2022.

The human *ADNP* gene, discovered in 1999 by Bassan et al. [33], spans ~40 kilobases and includes five exons and four introns with alternative splicing of an untranslated second exon. There is a striking degree of homology (90%) between human and mouse mRNA, and the region is highly conserved between vertebrates. The *ADNP* gene is located on the q13.13 band of chromosome 20 [34]. The *ADNP*-containing locus is frequently amplified in several cancers. Moreover, the down-regulation of *ADNP* by antisense oligodeoxynucleotides increases the expression of tumor suppressor p53 and decreases intestinal cancer cells’ vitality up to 90%, suggesting the involvement of *ADNP* in cell survival, probably through the modulation of p53 [34]. According to the Genotype-Tissue Expression (GTEx) database, the *ADNP* gene is found in central and peripheral nervous systems, as well as in different tissues and cells of various organs (Figure 2).

The *ADNP* gene encodes a protein containing nine zinc fingers, a homeobox domain and a bipartite nuclear localization signal, indicating transcription factor activity. The ADNP protein is mainly expressed in the cytoplasm of neuronal cells, whereas it is predominantly localized in the nucleus of non-neural cells [35,36]. Moreover, ADNP-like immunoreactivity was found in the extracellular milieu of astrocytes following stimulation with vasoactive intestinal peptide (VIP) [37].

ADNP, by interacting with nuclear chromatin, modulates the transcription of hundreds of genes involved in different biological events, such as embryogenesis [38], dendritic spine plasticity, autophagy, autism-linked protein translation and axonal transport [39]. Regarding the latter aspect, through the manganese (Mn^2+^)-enhanced magnetic resonance imaging technique, it was found that in *Adnp*+/+ mice, the signal intensity was significantly increased in the lateral part of the olfactory nerve and glomerular layer of the olfactory bulb. The signal intensity was significantly decreased in *Adnp*+/− mice, suggesting an alteration in the axonal transport [40,41].

*ADNP* is essential for brain formation and maintenance [42,43,44], and its expression is altered in different neurodegenerative diseases. In particular, the down-regulation of *ADNP* may concur with dopaminergic neurodegeneration in Parkinson’s Disease [45], and the *ADNP* plasma/serum and lymphocyte mRNA levels were correlated to clinical stage and Alzheimer’s disease (AD) biomarkers [46]. *ADNP* somatic mutations were found in the brains of AD-affected patients [47], as well as it being one of three genes frequently associated with autism spectrum disorders (ASD). The ADNP syndrome, also known as Helsmoortel–Van der Aa syndrome (HVAS), is characterized by a plethora of clinical symptoms, including global developmental delays, motor dysfunctions, hypotonia, and repetitive infections, as well as ophthalmic abnormalities, which suggest the multisystem nature of this disorder [48,49,50,51,52,53].

In 1999, a small peptide of eight amino acids derived from ADNP was synthesized, known as NAP (davunetide, NAPVSIPQ/Asn-Ala-Pro-Val-Ser-Ile-Pro-Gln) [33], and found to play a protective role at femtomolar concentrations [33]. The SIP motif in NAP interacts with the microtubule end-binding proteins, such as end-binding proteins 1 and 3 (EB1 and EB3), promoting microtubule intervention on neuroplasticity and neuroprotection [54,55]. In fact, EB3 plays a pivotal role in dendritic spine formation, and the positive effect of NAP in this process is EB3-dependent. Furthermore, in rat pheochromocytoma (PC12) cells and in rat cortical astrocytes, NAP treatment significantly increased the microtubule network area in the cell, an event preceding neurite outgrowth [56]. NAP is also involved in Tau–microtubule interaction, avoiding aberrant hyperphosphorylation and aggregation of Tau, which impairs cognitive functions [57]. Accordingly, *Adnp*+/− mice exhibited tauopathy features with a significant increase in phosphorylated Tau [48]. Moreover, NAP was found to enhance the autophagic process and preserve the cells against the accumulation of misfolded proteins, by promoting ADNP interaction with MAP1-associated protein 1 light chain 3 (LC3), representing the fundamental constituent of the autophagosome [58]. The chemical structure of this small fragment peptide allows it to enter the cells by dynamin-associated endocytosis [59], exerting protective effects, both in vitro and in vivo [60,61,62,63]. NAP protected neuronal-like cells against oxidative stress [64] and counteracted apoptotic cell death in neurons exposed to β-amyloid or tetrodotoxin treatment or glucose deprivation [65,66]. NAP ameliorated injury response in mice exposed to a closed head injury [67,68] whereas in a diabetes rat model NAP treatment partially rescued memory deficits by preventing the reduction of gray matter density [69]. Moreover, NAP intranasal treatment seems to exert moderate improvements on some cognitive deficits of schizophrenia patients [70,71].

Very recently, Karmon et al. [72] generated transgenic mice carrying the most common human p.Tyr719 (Tyr) *ADNP* mutation. This mouse model, as compared to the *Adnp*+/− model, showed greater severity of the phenotype due to heterozygous expression of 50% *WT-Adnp* (loss of function) and 50% *Tyr-Adnp* (potential gain of toxic function) alleles. For example, hyperphosphorylated tau deposits associated with visually evoked potential impairments were found in the hippocampus of ∼2-month-old *Tyr-Adnp* with respect to 11-month-old *Adnp*+/− male mouse brains [48]. Moreover, the *Tyr-Adnp* mice model reflects even more sexual dichotomy, as compared to *Adnp*+/− mice, as confirmed by the early developmental and motor delay in females, rather than males, with ASD [73].

## 4. The Role of ADNP in the Eye

ADNP shows widespread tissue and organ distribution. It has been detected in the brain, endocrine, respiratory, gastrointestinal and reproductive systems, as well as in skin, bone marrow and lymphoid tissues. In the eye, it was first reported in reference to its expression in the rat retina [1]. Here, the octapeptide NAP was shown to increase RGC survival after neurotrophic factor deprivation and to promote neurite outgrowth, confirming previous studies which demonstrated its ability to enhance neuronal survival and support axonal elongation [33,74,75]. Its pro-survival effects on RGCs were also displayed in vivo after retinal ischemia and optic nerve crush in rats after its intravitreal injection [76]. This immediate method of NAP administration also showed significant ameliorative effects of rat retinal damage after laser photocoagulation [77]. Moreover, stable transfection of NAP in rat retinal Müller cells exerted a protective role, not only in these cells against hypoxia-induced apoptosis, but also in other retinal neural cells, including neurons, astrocytes, and photoreceptors exerting nourishing effects against hypoxia-induced injuries [78]. Retinal diseases associated with hypoxia mainly include glaucoma, retinal ischemia and diabetic retinopathy [79,80]. The latter is characterized by vessel impairment induced by hyperglycemia, which contributes to the development of a hypoxic microenvironment in the retina [81,82]. The hypoxic event induces vascular-endothelial growth factor (VEGF) over release, responsible for aberrant neo-angiogenesis leading to blood–retinal barrier (BRB) breakdown [83]. Treatment with NAP was shown to keep the integrity of the outer BRB, counteracting human RPE apoptotic cell death induced by hyperglycemic/hypoxic insult [84]. NAP exerted these effects by modulating the expression of hypoxic inducible factors (HIFs). In particular, the peptide affected HIF-1α and HIF-2α expression, which, under hypoxia, elude the proteasome degradation system, translocating into the nucleus and triggering many target genes, including VEGF [85,86]. The ability of NAP to modulate key elements associated with hyperglycemic/hypoxic damage was also demonstrated in the retina of diabetic rats. In particular, a single intravitreal injection of NAP significantly reduced the expression of HIF-1α, HIF-2α, and VEGF [87]. It is well known that the hyperglycemic/hypoxic event also promotes the release of inflammatory cytokines, which concur with the impairment of BRB. NAP treatment was shown to modulate the inflammatory event during the early phase of DR. In fact, the intravitreal administration of NAP interfered with the expression of IL-1family members. Moreover, the peptide preserved outer BRB integrity after hyperglycemic–inflammatory insult in an in vitro model of DR [88].

**Figure 3:**
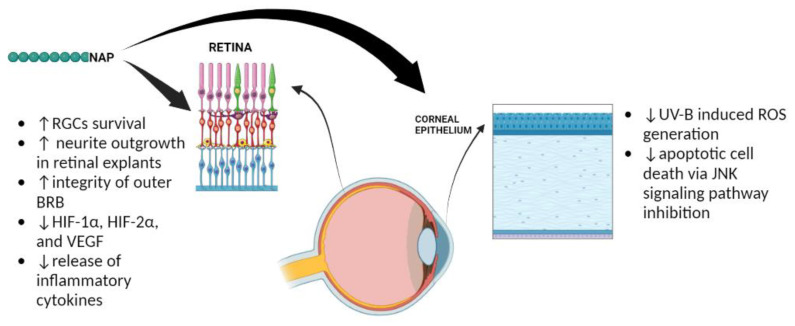
Protective effects played by NAP in the eye. The ↑ and ↓ refer to increase and decrease, respectively.

The expression of ADNP was also observed in human and rabbit corneal epithelium [2]. In particular, ADNP was mainly expressed in the basal layer that is characterized by limbal epithelial stem cells involved in the corneal epithelium regeneration [89]. This finding suggested a possible role of ADNP in corneal regeneration. Furthermore, NAP treatment of corneal epithelial cells exposed to UV-B radiations prevented ROS generation by reducing apoptotic cell death via JNK signaling pathway inhibition [2]. Overall, these data are summarized in Figure 3.

## 5. Perspective

Nowadays, there is an urgent need to identify innovative treatments to prevent and cure ocular diseases. The protective effects exerted by NAP in the eye are not a surprise, especially considering that HVDAS caused by *ADNP* mutations is also characterized by vision problems. In particular, the literature previously described the clinical case of two children, both carrying a nonsense mutation in the *ADNP* gene, showing intellectual disability and peculiar congenital eye anomalies [90], as well as another study which reported the case of an HVDAS-affected patient carrying a different *ADNP* gene mutation displaying convergent strabismus, astigmatism, hyperopia, unilateral iris coloboma and bilateral optic nerve coloboma [91]. In another paper, a detailed description of the ophthalmologic condition of an HVDAS-affected child bearing an *ADNP* gene mutation, was provided. This patient showed various eye dysfunctions mainly affecting the retina [92]. This evidence, thus, further supports the important protective and regenerative effects of *ADNP* in the eye. Considering that NAP treatment was previously approved in clinical trials for some pathologies, such as progressive supranuclear palsy (PSP), mild cognitive impairment (MCI), and schizophrenia [20,93], its use for the treatment of some ocular diseases can also be speculated.

## Data Availability

Not applicable.
